# Mental fatigue mediates the relationship between qi deficiency and academic performance among fifth-grade students aged 10–13 years

**DOI:** 10.3389/fpsyg.2024.1369611

**Published:** 2024-05-30

**Authors:** Xinzhu Wang, Xinyu He, Kaixian Fu

**Affiliations:** Educational department, Xichang University, Xichang, China

**Keywords:** qi deficiency, mental fatigue, academic performance, Chinese traditional medicine theory, fifth-grade students

## Abstract

**Background:**

Health has effects on children’s academic performance. Qi deficiency is generally used to assess an individual’s health in the Chinese traditional medicine theory. This study explores the effects of qi deficiency on children’s academic performance and examines whether mental fatigue mediates these effects.

**Methods:**

A total of 550 students aged 10–13 in fifth-grade were surveyed in a big primary school in Sichuan Province in November 2023 using paper-pencil-based questionnaires. Qi deficiency and mental fatigue were assessed, and exam scores in Chinese and Mathematics were recorded. Pearson’s correlation and linear regression analyses were used to test the mediation model and hypotheses.

**Results:**

The fifth-grade students had mild qi deficiency (M = 2.09) and a mild state of mental fatigue (M = 2.38) on a five-point Likert scale. The average exam scores in Mathematics and Chinese were 70.07 and 74.44 points out of 100, respectively. Qi deficiency was associated with Mathematics scores (*r* = −0.37, *p* < 0.01) and Chinese scores (*r* = −0.30, *p* < 0.01), and mental fatigue (*r* = 0.47, *p* < 0.01). Furthermore, mental fatigue was associated with Mathematics scores (*r* = −0.46, *p* < 0.01) and Chinese scores (*r* = −0.34, *p* < 0.01). Linear regression analyses showed that qi deficiency significantly predicted Mathematics scores (*β* = −0.26, *p* < 0.01), Chinese scores (*β* = −0.19, *p* < 0.01), and mental fatigue (*β* = 0.41, *p* < 0.01). When qi deficiency was controlled for, mental fatigue significantly predicted Mathematics scores (*β* = −0.28, *p* < 0.01) and Chinese scores (*β* = −0.17, *p* < 0.01).

**Conclusion:**

The mediation model and hypotheses were well supported, indicating that mental fatigue mediated the influence of qi deficiency on academic performance of fifth-grade students. Furthermore, the mediation effect of mental fatigue on Mathematics scores was a little stronger than that on Chinese scores.

## 1 Introduction

The health of children and their academic performance are the primary concerns within Chinese households. There is ample evidence supporting the influence of health on children’s academic performance. A systematic review revealed that the associations between physical fitness and academic performance in children and adolescents are weak to moderate (*β* = 0.10–0.42 and odds = 1.01–4.14) (Santana et al., 2017). A recent study utilizing a sample of 432 fifth-grade students in China has revealed a positive correlation between academic performance and physical fitness, as measured by upper limb, trunk, and lower limb strength indices (Xu and Sun, 2023). Height is considered a useful proxy for overall health in studies (Quanjer, 2023; Thompson and van Ophem, 2023), and a German study demonstrated that a 1 cm increase in height was associated with a 1.6% increase in the probability of attending the Gymnasium (the most academic secondary school track) (Cinnirella et al., 2011). The early-life stature has been demonstrated as a robust predictor for preschool cognitive development, schooling attainment, and cognitive functioning in adulthood (Stein et al., 2023). Mental health was also demonstrated to be correlated with academic achievement. A longitudinal birth cohort study involving 1,700 children revealed that mental health problems in early childhood and adolescence increase the risk for poor academic performance in schools (Agnafors et al., 2021). Impaired mental health status in the first semester of university study significantly predicts an increased incident risk of poor academic performance during the overall undergraduate period (Chu et al., 2023).

To date, however, few studies have explored the effects of qi deficiency on children’s academic performance. Qi deficiency serves as a crucial indicator for an individual’s health impairment, particularly in terms of physical well-being, and is generally used to assess individuals’ health in the Chinese traditional medicine theory (CTM) (Xue et al., 2020; Birch et al., 2022). Drawing on CTM and the literature on psychology, this study explores the association between qi deficiency and academic performance among fifth-grade students and highlights the theoretical and practical implications of qi deficiency in psychology.

In CTM, qi is one of the fundamental substances in the human body and is considered to provide energy and power to the body. Korngold and Beinfield (2006) suggested that qi is the refined nutrition and energy as well as their flows and transformations within the human body. Qi deficiency is a lack of body strength, indicating that the body is in a weak state (Chiang et al., 2012). Previous studies have demonstrated that qi deficiency correlates with reduced health and various health issues. A rat model demonstrated that qi deficiency is related to depression (Xiao-Juan et al., 2020). Qi deficiency and qi stagnation in both sexes, especially qi deficiency in males, are associated with major depression. Additionally, qi deficiency was associated with anxiety disorders in both sexes (Kondo et al., 2011). Generally, lack of energy and vitality, lassitude, and tiredness can be attributed to qi deficiency (Xing-Tai et al., 2015). Individuals with qi deficiency are very susceptible to various diseases or physical discomfort. For instance, qi-deficient children are more likely to get colds (due to qi deficiency in the lung) and indigestion (due to qi deficiency in the spleen and stomach). Qi deficiency in the kidney is regarded as the main cause of chronic kidney disease (Wang et al., 2022). In CTM, qi supplementation is a commonly used method to cure emotional disorders caused by qi deficiency. Supplementing qi in the spleen and soothing qi in the liver can effectively treat depressive symptoms (Ya-jing et al., 2022). A medical experiment demonstrated that using a special herbal extract granule prescribed to treat qi deficiency in the spleen can effectively alleviate physical discomfort and negative affect while promoting positive mood (Xue et al., 2012). To summarize, in CTM studies and clinical practice, qi deficiency serves as a very effective indicator of the individual’s overall compromised health status.

Mental fatigue is defined as a decline in the ability and efficiency of mental activities caused by excessive mental activities (Lambert et al., 2024). It is typically characterized by a decline in cognitive function, including the deterioration of visual search abilities, problem-solving skills, and attention allocation, ultimately leading to diminished cognitive performance (Brazaitis and Satas, 2023). In addition, mental fatigue is usually accompanied by feelings of tiredness, exhaustion, an aversion toward the present activity, or lack of energy, and can negatively affect health, well-being, and productivity (Brazaitis and Satas, 2023). Electroencephalography studies showed that mental fatigue is associated with abnormal brain activities, such as large increases in theta activity in frontal, central and posterior sites, and moderate changes in alpha activity in central and posterior sites.(Tran et al., 2020). Mental fatigue has been identified as a contributing factor to decreased academic performance in studenta. For instance, a study of 313 students revealed that mental fatigue is associated with reduced well-being and lower academic attainment in the university (Smith, 2018). A Danish study revealed that for every hour later in the day, children’s test scores decreased by 0.9% of a standard deviation due to increasing mental fatigue in daily learning (Sievertsen et al., 2016).

Chinese traditional medicine theory studies have demonstrated that qi deficiency can lead to several mental symptoms very similar to those associated with mental fatigue. By interviewing 26 patients with qi deficiency, CTM doctors observed that qi deficiency syndrome generally manifested as fatigue (physically), lassitude (mentally), laziness to speak, shortness of breath, spontaneous sweating, negative emotions, and decreased resistance (such as high susceptibility to catching a cold) (Li et al., 2017). A clinical study assessed 17 patients with kidney qi deficiency syndrome using various clinical tests and observed that these patients exhibited cognitive abnormalities in memory, executive function, and attention compared to their healthy counterparts (Dongqing et al., 2017). Another CTM study suggested that depression due to qi deficiency in the liver generally manifests as slow thinking and declined cognitive function (e.g., attention deficit, prolonged reaction time, learning difficulties, and poor language fluency) in addition to low mood and decreased motivation (Ming et al., 2016). Experimental studies with rat models also indicated that qi deficiency can lead to cognitive impairment. Jiu-lin and Zhi-hua (2018) reported that mild qi deficiency (as indicated by heart function) can lead to a decrease in learning ability in mice. Furthermore, Libin et al. (2011) observed worse memory and lower neural activities in qi-deficient (qi in spleen) rats compared to normal rats, and the impaired learning and memory abilities were recovered after treatment with Chinese herbs for regulating qi deficiency in the spleen. In conclusion, qi deficiency can cause mental fatigue.

Parents and teachers in China are deeply concerned about their children’s academic achievements in school. The purpose of this study is to examine the impact of physical health, specifically using qi deficiency as a proxy variable, on students’ academic performance and the underlying mechanisms involved. Based on the aforementioned literature review, this study postulates that qi deficiency, as an indicator of physical health, exhibits a close association with students’ academic performance. Furthermore, mental fatigue serves as a mediating variable in the relationship between qi deficiency and academic performance. Therefore, we proposed the following research hypotheses:

H1: Qi deficiency is negatively associated with academic performance among fifth-grade students.

H2: Qi deficiency is positively associated with mental fatigue in fifth-grade students.

H3: Mental fatigue mediates the relationship between qi deficiency and academic performance among fifth-grade students.

## 2 Research method

The current study was conducted according to the Declaration of Helsinki and was approved by the Research Ethics Committee of Xichang University (LG2021). Verbal informed consent was obtained from the parents or legal guardians of the participants.

### 2.1 Research design

The current study is based on cross-sectional data. We employed the convenience sampling technique to recruit samples from primary school students and utilized reliable scales to measure their qi deficiency and mental fatigue. We also collected their academic performance in school (i.e., the Mathematics and Chinese test scores in 1 week prior to this survey). Subsequently, linear regression analysis was conducted to investigate the mediating effect of qi deficiency. Finally, we discussed the verification results of the hypotheses and highlighted the significance, strengths, and limitations of the study.

### 2.2 Instruments

This study used six items to measure qi deficiency. These items were developed based on CTM studies (Chiang et al., 2012; Kim et al., 2016). Participants were asked to recall their physical sensations over the past 3 months and respond to five items using a five-point Likert scale ranging from 1 (never) to 5 (all the time). One of the items reads, “I feel bodily fatigued after mild activities.” The Cronbach’s alpha coefficient of this scale was 0.74, indicating high reliability. High mean scores on the scale indicated high levels of qi deficiency for students.

The present study used five items to assess mental fatigue among students. These five items were derived from the mental symptoms subscale in the 14-Item Fatigue Scale constructed by Chalder et al. (1993). Students were demanded to evaluate their mental status in the classroom over the past 2 months, focusing on aspects such as attention, memory, interest in courses, clarity of mind, and slips of the tongue in answering questions. All of these items were incorporated into a five-point Likert scale ranging from 1 (never) to 5 (all the time). One of the items reads, “I make slips of the tongue when answering questions in class.” The Cronbach’s alpha coefficient of this scale was 0.73, indicating high reliability. High average scores in the measurement indicated high levels of mental fatigue in school.

In the survey, participants are demanded to report their test scores in Mathematics and Chinese in 1 week prior to the survey. In primary school education in China, Chinese and mathematics are the two most important subjects, taking up the most class hours. Therefore, the scores of these two disciplines can well reflect the academic performance of fifth-grade students.

Due to the strict scrutiny and content restrictions of the schools in which the sample was located, only five demographic variables were included in the questionnaire, including age, gender, whether they were only children (WOC), ethnicity, and family housing conditions (FHC). Students were required to evaluate their FHC on a five-point scale, with “1” indicating “very poor” and “5” indicating “very good” compared to the housing condition of their neighbors. FHC is considered an valid indicator of family economic background in China (Wang et al., 2019) and has significant influence on children’s academic performance (Long et al., 2020).

### 2.3 Participants and context

This study employed convenience sampling to obtain samples from School A in Liangshan, Sichuan Province in China. School A is a large-scale middle school with an enrollment of over 8,000 students. All participants were selected from the fifth grade of this school. Prior to conducting the survey, informed consent was obtained from the parents or legal guardians of the students. The questionnaire was administered in paper format and distributed by psychology interns.

The survey was conducted within 1 week following a grade examination to ensure their scores were fresh in their memory. Prior to completing the questionnaire, students were explicitly informed that it was solely for research purposes and anonymity was guaranteed by not requiring them to provide their names. And they were also instructed to refrain from engaging in conversation with their peers while completing the questionnaire.

A total of 658 questionnaires were sent out, and 550 valid questionnaires were received. The effective rate was 83.59%. The sample characteristics are described in Table 1.

**Table 1:** Demographic characteristics of the sample (*N* = 550).

Variables levels		Frequency	Percentage
Age (years old)	10	158	35.11
	11	206	45.78
	12	61	13.56
	13	25	5.56
Sex	Male = 0	243	54.00
	Female = 1	207	46.00
Whether they are only child in family (WOC)	Yes = 0	66	14.67
	No = 1	384	85.33
Ethnicity	Han = 0	314	69.78
	Minority = 1	136	30.22
Family housing conditions (FHC)	Very poor	37	8.22
	Poor	62	13.78
	Average	96	21.33
	Good	92	20.44
	Very Good	163	36.22

### 2.4 Data analysis

In this study, the data will be analyzed using descriptive statistics, Pearson’s correlation, and linear regression techniques to investigate the mediating role of mental fatigue in the relationship between qi deficiency and academic performance.

## 3 Results

### 3.1 Means, standard deviations, and correlations

The descriptive statistics and correlations are presented in Table 2.

**Table 2:** Descriptive statistics and correlation analysis.

	M	SD	Qi deficiency	Mental fatigue	Mathematics scores	Chinese scores	FHC
Qi deficiency	2.09	0.75	1				
Mental fatigue	2.38	0.78	0.47^**^	1			
Mathematics scores	70.07	12.68	−0.37^**^	−0.46^**^	1		
Chinese scores	74.44	16.01	−0.30^**^	−0.34^**^	0.59^**^	1	
FHC	3.63	1.32	−0.29^**^	−0.30^**^	0.42^**^	0.42^**^	1

^**^p < 0.01. FHC refers to family housing conditions.

As indicated in Table 2, the mean score of qi deficiency is 2.09 on a five-point Likert scale ranging from 1 (never) to 5 (all the time). More precisely, this mean score falls between “occasionally” and “sometimes,” being very close to “occasionally.” The average score of mental fatigue is 2.38, lower than the middle value of 3.00 in the five-point Likert scale ranging from 1 (never) to 5 (all the time). Specifically, such an average falls between “occasionally” and “sometimes,” being very close to the middle point.

The average FHC score was 3.63 on the five-point Likert scale ranging from 1 (very poor) to 5 (very good), indicating that this average score falls between “average” and “good.” The average scores in Mathematics and Chinese were 70.07 and 74.44 points (out of 100), respectively.

The correlation coefficients are highly statistically significant. Qi deficiency was positively correlated with mental fatigue (*r* = 0.47, *p* < 0.01) and negatively correlated with Mathematics scores (*r* = −0.37, *p* < 0.01) and Chinese scores (*r* = −0.30, *p* < 0.01). Mental fatigue was negatively associated with Mathematics scores (*r* = −0.46, *p* < 0.01) and Chinese scores (*r* = −0.34, *p* < 0.01) (see Table 2).

### 3.2 Regression analysis

In this study, a three-step regression technique was employed to identify the mediating roles among the variables (Wen et al., 2004). Initially, a dependent variable (Y) was regressed on an independent variable (X); if the regression effect proved significant, we proceeded to the second step where the mediator variable (M) was regressed on X. Subsequently, when M is significantly predicted by X, Y was regressed simultaneously on both X and M in the third step to establish the mediation model. M is determined as a mediating variable by its ability to significantly predict Y in this final step. Our objective in this study was to demonstrate that mental fatigue mediates the relationship between qi deficiency and academic performance (measured by Mathematics scores and Chinese scores). The three steps of regressions include controlling variables such as sex, age, ethnicity, WOC, and FHC.

The results of regression analysis are presented in Table 3.

**Table 3:** Regression analysis results.

	Step1: Math scores		Step1: Chinese scores		Step2: Mental fatigue		Step3: Math scores		Step3: Chinese scores	
	*β*	*t*	*β*	*t*	*β*	*t*	*β*	*t*	*β*	*t*
(Constant)		8.61^**^		4.83^**^		2.40^*^		9.63^**^		5.27^**^
Qi deficiency	−0.26	−6.22^**^	−0.19	−4.37^**^	0.41	9.70^**^	−0.14	−3.27^**^	−0.12	−2.54^*^
Age	0.04	0.84	0.08	1.72	0.03	0.54	0.05	1.03	0.09	1.84
Sex	0.16	3.10^**^	0.20	4.75^**^	−0.06	−1.39	0.14	3.74^**^	0.19	4.56^**^
WOC	−0.13	−3.15^**^	−0.06	−1.51	0.12	2.78^**^	−0.10	−2.44^*^	−0.04	−1.05
Ethnicity	−0.12	−2.50^*^	−0.07	−1.42	−0.00	−0.02	−0.12	−2.61	−0.07	−1.44
FHC	0.31	7.19^**^	0.33	7.61^**^	−0.15	−3.53^**^	0.26	6.35^**^	0.31	7.01^**^
Mental fatigue							−0.28	−6.26^**^	−0.17	−3.56^**^
*F*	31.45^**^		62.03^**^		27.40^**^		34.87^**^		24.73^**^	
*Adj.R^2^*	0.29		0.25		0.26		0.35		0.27	

^*^p < 0.05, ^**^p < 0.01. WOC refers to whether they are only-child in family (Yes = 0, No = 1); FHC refers to family housing conditions. In sex, Male = 0, Female = 1. In ethnicity, Han = 0, minority = 1.

In step 1 of the regression analysis, we conducted separate regressions for Mathematics scores and Chinese scores with qi deficiency as the predictor. Qi deficiency significantly predicted Mathematics scores (*β* = −0.26, *p* < 0.01) and Chinese scores (*β* = −0.19, *p* < 0.01) (see Step 1 in Table 3), which supported our first hypothesis that qi deficiency is negatively associated with academic performance among fifth-grade students.

In Step 2, we regressed mental fatigue on qi deficiency. The results revealed that qi deficiency significantly predicts mental fatigue (*β* = 0.41, *p* < 0.01) (see Step 2 in Table 3), which supports our second hypothesis that qi deficiency is positively associated with mental fatigue in fifth-grade students.

Lastly, we regressed academic performance on qi deficiency and mental fatigue simultaneously. The results showed that mental fatigue could negatively predict Mathematics scores (*β* = −0.28, *p* < 0.01) and Chinese scores (*β* = −0.17, *p* < 0.01), and qi deficiency remained a significant predictor of Mathematics scores (*β* = −0.14, *p* < 0.01) and Chinese scores (*β* = −0.12, *p* < 0.05), although the *βs* became smaller than those in Step 1. Therefore, our third hypothesis was very well verified, indicating that mental fatigue mediates the association between qi deficiency and academic performance among fifth-grade students.

Regarding the association between controlling variables with mental fatigue, we found that (1) WOC was associated with mental fatigue positively (*β* = 0.12, *p* < 0.01), indicating that students with one or more siblings in the family had higher levels of mental fatigue than those without any siblings; (2) FHC was associated with mental fatigue negatively (*β* = −0.15, *p* < 0.01), indicating that the students from poor families had higher levels of mental fatigue than those from rich families.

Regarding the association between controlling variables with academic performance, we found that (1) sex was associated positively with Mathematics scores (*β* = 0.14, *p* < 0.01) and Chinese scores (*β* = 0.19, *p* < 0.01), indicating that girls tend to outperform boys in academic performance; (2) WOC was associated negatively with Mathematics scores (*β* = −0.10, *p* < 0.05), indicating that students without any siblings had higher Mathematics scores than those with one or more siblings; (3) FHC was associated positively with Mathematics scores (*β* = 0.26, *p* < 0.01) and Chinese scores (*β* = 0.31, *p* < 0.01), indicating that the students with good family housing conditions had better academic performance than those with poor family housing conditions.

**Figure 1:**
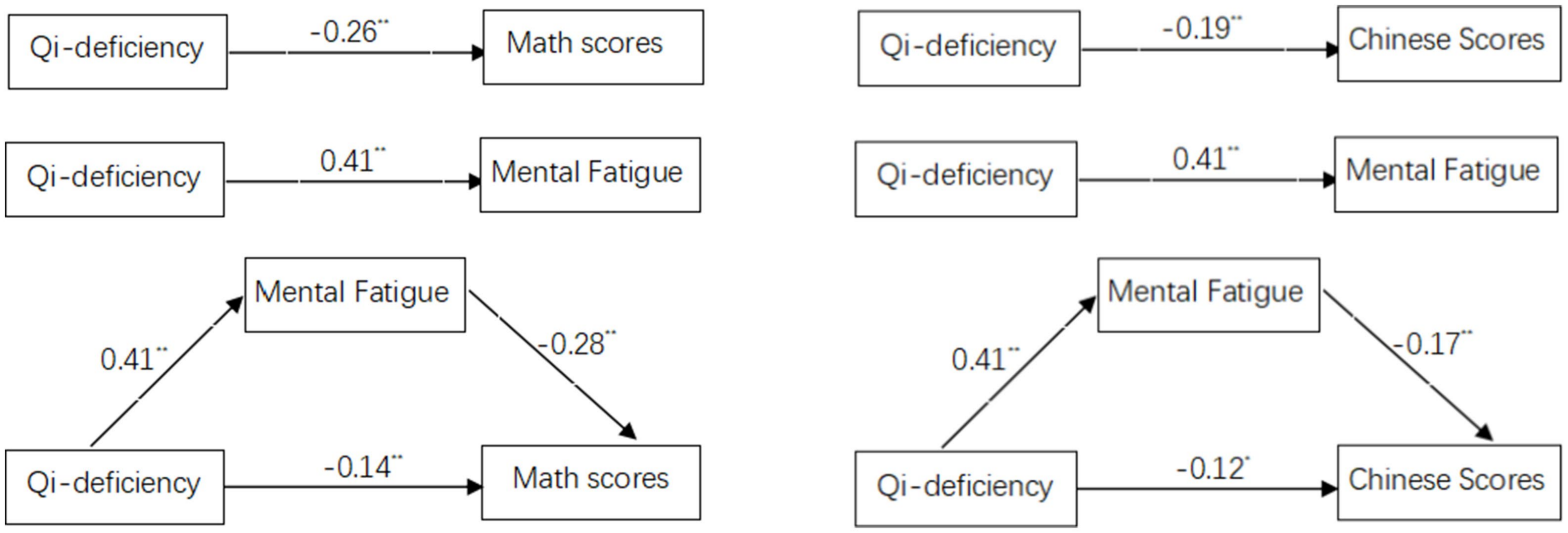
The meditation effects of mental fatigue.

Figure 1 illustrates the mediation effects of mental fatigue in the association between qi deficiency and academic performance. A stronger association was noted between qi deficiency and Mathematics scores (*β* = −0.26, *p* < 0.01) than that between qi deficiency and Chinese scores (*β* = −0.19, *p* < 0.01). Similarly, a stronger association was observed between mental fatigue and Math scores (*β* = −0.28, *p* < 0.01) than that between mental fatigue and Chinese scores (*β* = −0.17, *p* < 0.01). The indirect effect of qi deficiency on Mathematics scores is 0.115 (i.e., 0.41*0.28), accounting for 44.15% (i.e., 0.115/0.26) of the total effect of qi deficiency on Mathematics scores. The indirect effect of qi deficiency on Chinese scores is 0.070 (i.e., 0.41*0.17), accounting for 36.68% (i.e., 0.070/0.19) of the total effect of qi deficiency on Chinese scores. Therefore, the mediation effect of mental fatigue in the association between qi deficiency and Mathematics scores was a little greater than that between qi deficiency and Chinese scores (see Figure 1).

## 4 Discussion

### 4.1 Descriptive statistics for qi deficiency, mental fatigue, and academic performance

In this study, the average score for qi deficiency is 2.09 on a five-point Likert scale, suggesting that the fifth-grade students experience occasional somatic symptoms of qi deficiency. Therefore, we conclude that overall, fifth-grade students are in a mild qi deficiency status.

The average score for mental fatigue is 2.38 on a five-point Likert scale, suggesting that fifth-grade students generally experience a state of mild mental fatigue. This indicates that on the whole fifth-grade students maintain a positive mental state in the classroom.

The mean scores for Mathematics and Chinese were 70.07 and 74.44 points (out of 100), respectively, indicating the challenging nature of these exams for fifth-grade students.

### 4.2 Relationship between qi deficiency, mental fatigue, and academic performance

Our first hypothesis, asserting that qi deficiency is negatively associated with academic performance among fifth-grade pupils, is strongly supported by the results of the regression analysis in Step 1 (see Table 3). Qi deficiency is a useful construct that reflects an individual’s state of physical health in traditional Chinese medical practice. Therefore, such a finding in this study echoed the views in previous studies that students’ physical health has effects on their academic outcomes, and better physical health is linked to better grades (Van Dusen et al., 2011; Santana et al., 2017).

The regression analysis in Step 2 in Table 3 robustly supported our second hypothesis that qi deficiency is positively associated with mental fatigue among fifth-grade pupils. This finding is consistent with the viewpoints in CTM studies by Ming et al. (2016), Dongqing et al. (2017), and Li et al. (2017). They reported that individuals with qi deficiency exhibit a range of cognitive symptoms, such as mental lassitude, declined memory, decreased executive function, attention deficit, prolonged reaction times, and poor language fluency. Importantly, these symptoms are consistent with or closely resemble the manifestations of mental fatigue. According to CTM, qi represents the refined nutrition and energy as well as their flows and transformations within the human body. Qi deficiency indicates a lack of nourishment and energy for the brain, resulting in declined mental function. Therefore, qi deficiency is positively associated with mental fatigue.

Our third hypothesis, stating that mental fatigue mediates the relationship between qi deficiency and academic performance among fifth-grade pupils, is well supported. Firstly, qi deficiency had a positive impact on mental fatigue but a negative impact on academic performance. Secondly, with qi deficiency and the controlling variables entering into the regression model, mental fatigue could significantly and negatively predicted Chinese and Mathematics scores (see Step 3 in Table 3), demonstrating the mediation role of mental fatigue. A negative correlation between mental fatigue and academic performance was also demonstrated by Smith (2018), who reported that college students with high mental fatigue had reduced well-being and lower academic attainment compared to those with low mental fatigue.

This study observed that the mediation effect of mental fatigue was a little greater in the relationship between qi deficiency and Mathematics scores compared to that between qi deficiency and Chinese scores (see Figure 1). This finding may be because Chinese is the mother tongue of all the children in this study and is easier to learn compared to Mathematics, so the Chinese test scores are less affected by mental fatigue. Mathematics is more difficult to learn than Chinese for Chinese students, as evidenced by math anxiety—a common phenomenon among Chinese students (Ching, 2017).

### 4.3 Effects of controlling variables

Whether they were only children was associated with mental fatigue and Mathematics scores in the present study. Specifically, the only-child group had lower levels of mental fatigue and higher Mathematics scores than the non-only-child group. According to the resource dilution model, the more children there are in a family, the fewer resources each child can enjoy, which in turn affects their acquisition of human capital (Öberg, 2017). In short, the availability of resources leads to better mental state and academic performance among children who have no siblings.

Family housing condition was associated with mental fatigue and academic performance. Specifically, the students with better family housing conditions had lower mental fatigue and better academic performance than their counterparts with poor family housing conditions. Generally, families with better housing conditions are richer, and they can provide their children with a better growth environment and more educational resources. Thus, their children will be healthier and have better academic performance (Hin Li, 2012).

Furthermore, sex was associated with academic performance, and girls had higher scores than boys in Chinese and Mathematics. There are two reasons for this. First, in Chinese primary schools, girls are more self-disciplined than boys, which gives the girls an edge in learning (Duckworth and Seligman, 2006). Second, in general, boys are less study-oriented than girls (Houtte, 2004).

### 4.4 Theoretical and practical implications

This study enriches the understanding of the effects of physical health on children’s academic performance. Unlike other indicators of physical health, including height and physical fitness, the concept of qi deficiency stems from CTM, a very old and widely acknowledged theory. According to CTM, qi is the fundamental substance that provides energy and power for physical and mental activities. Qi deficiency implies that there is insufficient energy and power to support intellectual activities. Studies in CTM have demonstrated that severe qi deficiency can lead to cognitive impairment (Dongqing et al., 2017; Li et al., 2017). In this study, children in grade 5 were in a mild state of qi deficiency, indicating that they were slightly weak in body but far from being sick. However, the effects of physical health on academic performance were still observed clearly among these children.

Furthermore, this study makes a valuable contribution to the shared areas between CTM and health psychology. CTM holds that the state of qi and emotions are interrelated and interacted, suggesting that the state of qi can impact emotions, and vice versa. Previous CTM studies have demonstrated that qi deficiency leads to emotional problems such as anxiety and depression (Kondo et al., 2011), and this study established the association between qi deficiency and mental fatigue. Qi deficiency can partially explain how some mental problems occur and suggests how to help cope with these mental problems using CTM methods.

This study has three practical implications. First, the association between qi deficiency and mental fatigue implies that addressing qi deficiency might alleviate mental fatigue. According to CTM, several methods can be used to address qi deficiency, including practicing abdominal breathing (to complement qi in the lung), strengthening nutrition (to complement qi in the spleen and stomach), or massaging acupuncture points (to enhance the flow of qi). Therefore, there are several CTM approaches to alleviate mental fatigue. In short, the concept of qi deficiency provides a new perspective to study how to alleviate mental fatigue among students.

Second, this study reminds teachers to encourage children to carry out physical exercise during the 10-min break between classes. Currently, many primary schools demand their pupils to sit quietly in the classroom (unless going to the bathroom) to prevent them from getting physically injured during the 10-min break activities.^1^ According to CTM, physical activities and short rest can adjust the state of qi in the body, including the recuperation of qi; and psychology studies have revealed that short-term physical activity can effectively alleviate mental fatigue caused by prolonged learning, thereby improving cognitive function (Jacquet et al., 2021; Nezhad et al., 2022). Therefore, teachers should encourage children to engage in more activities during recess to restore qi and alleviate mental fatigue.

Third, this study helps change parents’ wrong viewpoint that physical conditions (as long as they are not clinical diseases) cannot affect children’s mental health and academic performance. This study indicates that mild qi deficiency (far from being a disease in the body) is significantly associated with mental fatigue and academic performance among fifth-grade students. Therefore, parents need to pay more attention to their children’s physical health, rather than over-encouraging them to learn hard.

## 5 Strengths and limitations

The first strength of the present study lies in its solid theoretical foundation (i.e., CTM). Qi deficiency, one of the main concepts in CTM, is considered a useful mechanism to explain the development of emotional problems and cognitive impairment. CTM studies have demonstrated that mental diseases and cognitive impairment can be alleviated or even cured by addressing qi deficiency (Aung et al., 2013; Jia-xi et al., 2023). Therefore, the association between qi deficiency and academic performance is well supported in solid theory. The second strength is that the current study demonstrates the potential interrelation between CTM and psychological status. Specifically, this study notes that qi deficiency is associated with mental fatigue among fifth-grade children. Looking at psychological phenomena from the perspective of CTM will make modern psychology more culturally inclusive.

There are two limitations associated with this study. First, using cross-sectional data merely establishes a correlation between qi deficiency, mental fatigue, and academic performance. Caution should be exercised in inferring a causal link between these variables. Second, this study used a convenience sample consisting of individuals aged 10 to 13 years, which may not represent children across all primary school years. Therefore, this study cannot be generalized to the entire population of children in primary schools. Future research must overcome these limitations to explore the relationship between qi deficiency and academic performance among children.

## 6 Main findings of this work

Based on CTM, this study investigates the association between qi deficiency, mental fatigue, and academic performance among fifth-grade students. The main findings echo the results in previous research, revealing that (1) Qi deficiency is significantly negatively correlated with academic performance; (2) Qi deficiency is positively associated with mental fatigue; and (3) Mental fatigue serves as a mediating factor in the relationship between Qi deficiency and academic performance. In addition, this study indicates that fifth-grade students in primary school exhibit mild symptoms of Qi deficiency and mild mental fatigue. This study enriches the understanding of the effects of physical health on children’s academic performance.

## Data availability statement

The raw data supporting the conclusions of this article will be made available by the authors, without undue reservation.

## Ethics statement

The studies involving humans were approved by the Research Ethics Committee of Xichang University (LG2021). The studies were conducted in accordance with the local legislation and institutional requirements. Written informed consent for participation in this study was provided by the participants’ legal guardians/next of kin.

## Author contributions

XW: Conceptualization, Writing – original draft, Writing – review & editing. XH: Methodology, Writing – review & editing. KF: Investigation, Methodology, Writing – review & editing.

## Footnotes

^1^In China, there were many cases in which children were bodily injured (mildly, severely, or even to death) during the 10-min break activities or physical exercise class. In these cases, schools had to pay huge compensations to their families, and some teachers were also implicated. Therefore, several primary schools began to restrict children’s free activities on campus.
